# Predictors of HIV testing and status disclosure among young adolescents in postconflict settings: findings from a pre–post study design in Nimule per-urban town of South Sudan

**DOI:** 10.1136/bmjopen-2024-094008

**Published:** 2025-04-02

**Authors:** Samuel Bojo, Gilbert Kokwaro, Ambrose Agweyu

**Affiliations:** 1 Strathmore University, Nairobi, Kenya; 2 Center for Research and Development in Population Health, Juba, Central Equatoria, South Sudan; 3 London School of Hygiene & Tropical Medicine Centre of Global Change and Health, London, UK; 4 KEMRI-Wellcome Trust Research Programme, Kilifi, Kenya

**Keywords:** Adolescents, Primary Prevention, Sexually Transmitted Disease, Adolescent

## Abstract

**Abstract:**

**Objective:**

To assess HIV testing and status disclosure rates and explore their associated predictors among young adolescents (10–17 years) who received health education through the Orphans and Vulnerable Children programme in Nimule, South Sudan.

**Design:**

A pre–post evaluation study with data collected at baseline (December 2020) and at the endline (December 2022).

**Setting:**

The study was conducted in Nimule, a densely populated periurban town characterised by high HIV prevalence and substantial cross-border movement between Uganda and South Sudan, making it a relevant setting for an HIV prevention project.

**Intervention:**

The primary intervention was HIV risk education delivered through forty peer-led health clubs. Adolescents were screened for HIV risk factors and referred for HIV and other sexually transmitted infection testing at health facilities.

**Participants:**

The study included young adolescents aged 10–17 years recruited from HIV-affected households within 17 neighbourhoods in Nimule periurban town. Informed consent was obtained from both caregivers and adolescents.

**Primary and secondary outcome measures:**

The primary outcome was self-reported HIV testing and status disclosure. Binary logistic regression was used to assess the association between the study outcome variables and associated sociodemographic factors.

**Results:**

A total of 557 (73.0%) of the 768 enrolled adolescents were surveyed at baseline and endline, including 301 (54.0%) females and 276 (46.0%) males. The median age was 14 years (IQR: 11–16) at baseline and 15 years (IQR: 12–17) at endline.

HIV testing increased from 315 (56.7%) at baseline to 557 (100%). The odds of undisclosed HIV status were 49% lower at endline adjusted OR (aOR) 0.51 (95% CI 0.92, 0.67; p<0.001). Status disclosure was nearly universal, with 557 (100%) at baseline and 556 (99.8%) at endline. Male adolescents aOR 0.77 (95% CI: 0.59, 0.99; p<0.04) and those cared for by their siblings or other relatives were less likely to know their HIV status (aOR 0.59; 95% CI: 0.41, 0.84; p<0.003).

**Conclusions:**

Male gender and being cared for by siblings or other relatives were strong predictors of lower HIV testing and status disclosure. These findings underscore the importance of targeting efforts towards this category of adolescents in postconflict and resource-limited settings like South Sudan while leveraging peer-led HIV education interventions in conflict-affected settings.

**STRENGTHS AND LIMITATIONS OF THIS STUDY**

- Use of standardised and pretested survey questionnaire.
- Training and supervision of data collection.
- Confounding factors accounted for in the analysis.
- Loss to follow-up and non-response of some participants could have affected the final study findings.
- Potential of social desirability bias where inaccurate responses from participants could have affected our findings.

## Introduction

 Globally, adolescents are vulnerable to HIV infections and frequently lack access to testing services.^1^ In South Sudan, only 39% of the population know their HIV status, despite a national generalised HIV prevalence of 1.9%,^2^ highlighting a wide gap in the HIV prevention cascade. In 2017, the Ministry of Health (MOH), with support from the US Presidency Emergency Plan for AIDS Relief (PEPFAR), conducted an HIV biobehavioural survey among female sex workers in Nimule. The survey revealed a high HIV prevalence of 23.2% with a disproportionately low level of HIV prevention awareness at 65.7%.^3^

Children continue to bear a substantial burden of the HIV epidemic in South Sudan. Out of 160 000 people living with HIV in 2023, 15 000 (9%) were children under 15 years.^2^ Among those living with HIV, only 51% knew their HIV status, and an estimated 1200 children aged 0–14 years were newly infected. The 2022 HIV programme review identified several challenges affecting Early Infant Diagnosis and treatment of children living with HIV, resulting in low uptake of the services and poor retention in care. Barriers included high levels of stigma and discrimination in both health facilities and communities, and gender-based violence from spouses after disclosure. In addition, children depend on their caregivers for accompaniment to HIV care and treatment facilities, and young children rely on caregivers to administer their medication.^4^

A 2022 qualitative study conducted among community care workers and providers examining continuity of treatment among Orphans and Vulnerable Children (OVC) found a lack of continuity of care between primary and secondary or substitute caregivers. Substitute caregivers were not always informed of the children’s status or where their medication is stored.^5^ Interventions aimed at improving access to and retention in care for children must, therefore, target caregivers, households and health providers with innovative behaviour change strategies.

In South Sudan, local organisations contribute substantially to HIV service delivery, demand creation and advocacy at the community level. Their deep community engagement uniquely positions them to support changes in attitudes and norms that hinder the uptake of HIV services, including for children. Therefore, they are critical in the localisation agenda. This leaves a wide gap in the prevention cascade among high-risk populations. Placing adolescents at the forefront of the fight against HIV and AIDS has been prioritised,^6^ yet South Sudan faces challenges in providing adolescents with access to HIV testing. While civil society organisations and international partners provide community-level services, most health facilities in South Sudan lack adolescent-friendly services, leading many adolescents to seek care in the private sector.^7^ South Sudan has developed several policy instruments to guide its national HIV response. These, among others, include national HIV policy,^8^ a Reproductive, Maternal, Newborn, Child, Adolescent Health and Nutrition Strategic Plan,^9^ a 5-year national strategic plan to guide its national HIV response,^10^ a national HIV prevention strategy,^11^ and a social and behavioural change communication policy.^12^ These documents prioritise scaling intervention targeting adolescents and young people. The United National Population Fund and US PEPFAR implementing partners such as IntraHealth International and JHPIEGO have been supporting the South Sudan MOH in implementation to reach adolescent girls and young women. However, despite these efforts, the introduction of pre-exposure prophylaxis services in 2021 was primarily targeted towards key populations in urban towns, leaving adolescents in the general population with limited access.

Unlike other countries in the region where the age of consent for HIV testing has been lowered to 14 years, the South Sudan MOH requires parental or caregiver consent for adolescent minors aged 10–14 years. However, emancipated minors aged 15–17 years can provide consent independently. With limited access to HIV testing services, this age restriction further limits access to vital testing services among adolescents. There are many platforms within HIV prevention programmes designed to expand HIV testing and status disclosure among adolescents, especially girls. Among these are the Determined, Resilient, Empowered, Aids-free, Mentored and Safe and OVC Programme. Recent studies have identified the OVC platform as a catalyst in closing critical gaps in HIV prevention and treatment outcomes among adolescents.^13^ The OVC programme is designed to serve adolescents who are living with or affected by HIV and AIDS (who have lost one or both parents to HIV and AIDS). Over the last decade, the US PEPFAR has committed about 10% of its budget to South Sudan to support OVC programmes in delivering impactful interventions for millions of children and adolescents impacted by HIV/AIDS.^14^ The uniqueness of the OVC programme in increasing HIV status disclosure is its ability to work with caregivers and clinical staff to deliver family-centred services to adolescents in their households based on their unique needs.^15^ This has, in turn, been observed to significantly increase the uptake of HIV testing services, status disclosure, retention on treatment retention and viral load suppression among adolescents in South Africa^16^ and Kenya.^17^ Moreover, a higher frequency of home visits, participation in support groups such as positive parenting and better economic status were observed to increase status disclosure and retention in ART by 85% among OVC in Tanzania.^18^ Unlike other countries, South Sudan’s conflict-affected setting with its unique challenges such as limited access to HIV prevention services, high population movement and high illiteracy level was the motivation for conducting this study.

This study aimed to assess HIV testing and status disclosure rates and explore their associated predictors among young adolescents (10–17 years) who received health education delivered in the OVC programme in Nimule, South Sudan.

## Materials and methods

### Study design

We conducted a pre–post evaluation with surveys at baseline (December 2020) and at the endline (December 2022) to collect data on self-reported HIV testing and status and associated demographic variables. Adolescents from HIV-affected backgrounds were recruited in peer-led health clubs where they completed health education, including receiving HIV risk screening tools and referral for testing in health facilities. In addition, their caregivers received parenting training and financial literacy training.

### Setting

The study was conducted in Nimule, a periurban town of South Sudan, which is located at the border between Uganda and South Sudan. The proximity of Nimule, a periurban town to the Ugandan border, makes it a refuge for thousands of internally displaced persons, leading to high population density and increased cross-border movement. Biobehavioural studies have revealed a high HIV prevalence among female sex workers in the area.^19^ Given the increased risk of HIV and other sexually transmitted infections among adolescents, this location was deemed suitable for this HIV prevention study.

### Intervention

We undertook a multifaceted intervention delivered using the OVC case management platform, where adolescents’ primary needs were assessed and intervention delivered based on their stratified individualised needs. Here, we used trained peer educators to deliver HIV risk education adapted from the South Sudan comprehensive sexuality education curriculum. Equally, their primary caregivers received parenting support training and financial literacy training and were provided business startup capital of US$200 disbursed in four quarterly payments of US$50 in 2021.

### Participants

The study recruited young adolescents aged 10–17 years from four hundred HIV-affected households within seventeen neighbourhoods in Nimule periurban town. We considered adolescents from HIV-affected households for their study because of their increased vulnerability to HIV, as some of them were living with HIV, for those who did not know their HIV status, providing targeted health education combined with referrals for HIV testing increased their chances of acquiring HIV testing and status disclosure.

### Primary and secondary outcome measures

The primary outcome of this study was self-reported HIV testing and status disclosure to any trusted individual (caregiver, siblings or peer). Independent variables included age, gender, education and type of caregiver. Data collection took place at two points at baseline (December 2020) and endline (December 2022).

#### Eligibility criteria

Adolescents eligible for inclusion in the study were those aged between 10 and 17 years, living in an HIV-affected household in Nimule town. Participation required assent from their caregiver, as well as a willingness to attend all health education sessions. Additionally, adolescents had to be living with a primary caregiver, with both agreeing to participate in the study.

Exclusion criteria included being younger than 10 years or older than 17 years, not residing in an HIV-affected household in Nimule town, unwillingness to participate in the study or the absence of a primary caregiver.

### Sample size estimation

We used the WHO (1991) cluster sampling strategy^20^ for estimating a population proportion with specified relative precision. Assume 50% of adolescents at baseline tested for HIV and disclosed their HIV status, confidence level of 95% and a design effect of 2, the estimated sample size was 768 adolescents. Given the postconflict setting with high population movement, we anticipated a 15% attrition or refusal rate.

### Sampling procedures

Adolescents were purposively recruited from households enrolled on the OVC programme. These households were first identified from the antiretroviral treatment therapy (ART) clinic in Nimule Hospital for people living with HIV on treatment. Trained case workers (research assistants) subsequently visited these households and recruited adolescents aged between 10 and 17 years after obtaining informed consent from both the caregiver and adolescents. This procedure was repeated until a total of 768 adolescents were recruited into the study from the 400 households.

### Study procedures

Adolescents were assigned to 40 health clubs, each facilitated by a trained Peer educator. Before the first session, a baseline assessment was conducted using a survey questionnaire to obtain information on participation in sociodemographic factors and self-reported HIV testing and status disclosure. Using a standardised HIV health education manual adopted from the South Sudan MOH, peer educators delivered HIV risk education in their clubs for 4 months, with each group meeting twice a month. Members were recruited voluntarily and with consent from their caregivers. Supported by their peer educators, group members were consulted and decided on their meeting time, date and venues, which were all suitable for them.

Peer educators used various interactive approaches including short presentations (30–40 min), followed by question-and-answer sessions, and breakaway sessions with games, plays, singing and storytelling to enhance engagement. Using the HIV risk screening tool (online supplemental appendix 2), peer educators screened the participants for risk of HIV (ever had sex), referred them for HIV testing and encouraged them to disclose their test results to trusted individuals. Additionally, caregivers were engaged in positive parenting and Village, Lending and Savings Association groups, where they received US$50 quarterly for the first year as seed capital. These interventions were aimed at empowering caregivers in providing nurturing, responsive parenting and confidently discussing sexuality education, generating income and meeting the cost of basic needs such as education, healthcare and food, as well as their capacity to manage HIV status disclosure among adolescents. After 2 years and the closure of the OVC programme, an endline survey was conducted using a standardised survey questionnaire (online supplemental appendix 1). We measured rates of self-reported HIV testing by asking adolescents at baseline and endline whether or not they had ever tested for HIV and what their test results were.

### Patient and public involvement

The study held stakeholder engagement meetings at the national and state levels involving the MOH, South Sudan HIV and AIDS Commission and partners to discuss the indicators to be measured, including the selection of study sites and targeted adolescent groups. This guided the choice of the indicators, target age groups and study site. In addition, caregivers and adolescents were also consulted to decide on how they wanted to get involved in the study activities, expectations and what their contribution would be to the study. This helped in identifying the appropriate meeting times, venues and dates for caregivers and adolescents.

### Data management

No identifiable information, aside from the voices of participants, was recorded. Each participant was assigned a unique subject ID number to assist the study team in tracking notes. Data were reported only in aggregate form, and personal identifiers were not collected. All notes and paper-based materials were securely stored in locked cabinets at the study sites, accessible only to investigators, research assistants and data analysts. After data collection, paper forms were stored securely and transferred to the OVC programme Management for storage within 60 days. Data were archived at the Head Office of the Centre for Research and Development in Population Health (CRDPH) in Juba. Disposition of confidential HIV information would be done by the designated Monitoring and Evaluation (M&E) team according to the MOH data disposition guideline. The Unique Personal Identifier Code was securely kept by the PI and not shared with anyone outside the study team.

### Quality control/assurance

Routine survey monitoring and close field supervision were conducted by supervisors, data managers and coinvestigators to ensure that the survey team did not deviate from the survey protocol and standard operating procedures and to help maintain data integrity. The following measures were undertaken to safeguard data integrity and mitigation of biases:

To minimise transcription errors, the principal investigator (PI), research coordinator and M&E officer provided training and technical supervision during data collection and reviewed data completeness and consistency. The M&E officer conducted a data quality assessment by verifying 10% of questionnaires with respective respondents before data entry and exporting to STATA for analysis. In addition, incomplete data due to non-responses or participant transferring out of the study area were removed from the endline dataset before analysis was performed.

To prevent enrolment of ineligible participants, research assistants were trained on identification and enrolment based on eligibility criteria with close supervision from the M&E officer.

Non-responses, due to refusal to answer all or part of the questionnaire and transfer out of the study area, were anticipated to occur. To account for this, the sample size was adjusted by 15%.

Awareness of the study objectives and research question by the research team would likely influence the alteration and omission of participants’ responses to the questionnaire. To mitigate this bias, the study objectives and research questions were concealed from data collectors. In addition, data collection at both the baseline and endline was done by different groups of data collectors. Additionally, the PI was not directly involved in the day-to-day implementation of planned activities and data collection.

### Data ownership, storage, sharing and release

A data governance document outlined the rights of each investigating institution regarding data, the roles and responsibilities of each partner for data stewardship, the membership of the data governance body, and the process by which this body would communicate decisions on data handling and access. The governance body approved the data management plan, granting the release of data and certifying the finalisation of data at the end of the survey. The data governance agreement covered the period from the start of survey data collection to the release of the survey report and dataset-associated documentation. The CRDPH implementing the programme was the primary owner of the data, having real-time, unimpeded access to the de-identified data for data monitoring, data analysis and report/manuscript development, as well as all coinvestigators.

### Public access

Deidentified data were owned by CRDPH and co-owned by the MOH under a written agreement. Data were made available to the researchers and subject to an approved concept sheet that has been reviewed and cleared by the data study oversight committee. Researchers who did not participate in the survey would be granted access to deidentified data sets, survey documentation, code books and questionnaires upon reasonable request to survey investigators.

### Data analysis

Data were captured in Microsoft Excel and exported into STATA V.16 for cleaning and analysis (online supplemental file 4). The datasets were merged to enable comparisons at baseline and endline. Descriptive statistics were used to summarise the sociodemographic characteristics of the study populations at baseline and endline.

### Exploratory analysis and statistical assumptions

We assessed the distribution of the data using histograms and the Shapiro-Wilk test for normality. We also tested for multicollinearity among independent variables using correlation matrices and the variance inflation factor.

### Univariate and bivariate analysis

Univariate analysis described the distribution of our variables across baseline and endline. McNemar’s test was used to examine changes over time for paired data. For associations between sociodemographics and outcomes, we used the χ^2^ test for categorical data.

### Multivariate analysis

To examine independent associations, we built multivariate models using a stepwise approach, including variables associated with the outcome at p<0.25 from the bivariate analysis. The Akaike information criterion (AIC) was used to select the most appropriate model, with lower AIC values indicating a better fit. Adjusted ORs (aORs) and corresponding p values (with p<0.05 considered statistically significant) were reported to represent findings from the multivariate analysis.

### Human subjects’ considerations

#### Potential risks

There were minimal physical risks for participants recruited into this study. A primary ethical concern of this survey was that participation in the survey may lead to other people discovering that participants are engaging in stigmatised behaviours such as sex, disclosure of HIV results and alcohol or drug use. To minimise these potential harms, we trained interviewers on ethical compliance. The survey did not collect any personal identifying information; all survey staff signed an assurance of confidentiality agreement.

## Results

### Participants’ sociodemographic characteristics

Of the 768 adolescents enrolled from 400 households, 211 (27.5%) were lost to follow-up primarily due to displacement caused by conflict, leading them to flee to Uganda as refugees. This reduced the total number of participants to 557 (73.0%) at the endline survey. More than half of the adolescents, 301 (54%) were female. The median age was 14 (IQR 11–16) at baseline with over 76% of adolescents enrolled in school both at baseline and endline survey. The majority, 462 (82.9%), were cared for by their parents with none reporting being married. Additionally, a substantial proportion, 430 (77.2%), reported having worked for pay in both cash and in-kind (table 1).

**Table 1:** Sociodemographic characteristics of respondents at baseline and endline (n=557)

Indicator	Baseline**n (%**)	Endline**n (%**)
Gender		
Female	301 (54.0)	301 (54.0)
Male	256 (46.0)	256 (46.0)
Age group		
10–14 years	328 (58.9)	266 (47.8)
15–17 years	229 (41.1)	291 (52.2)
Median age (IQR) years	14 (11–16)	15 (12–17)
Education		
None	7 (1.3)	7 (1.3)
Primary	427 (76.7)	411 (73.8)
Secondary	123 (22.1)	116 (20.8)
Tertiary	0 (0.0)	23 (4.1)
Reading proficiency		
Cannot read at all	99 (17.8)	99 (17.8)
Can partly read	174 (31.2)	174 (31.2)
Read a whole sentence	284 (51.0)	284 (51.0)
Owns a phone		
Yes	121 (21.7)	121 (21.7)
No	436 (78.3)	436 (78.3)
Current marital status		
Never been married	557 (100.0)	557 (100.0)
Employment status		
Employed	430 (77.2)	430 (77.2)
Unemployed	127 (22.8)	127 (22.8)
Health rating		
Poor/fair	242 (43.3)	232 (41.6)
Good	184 (32.9)	308 (55.2)
Excellent	131 (23.8)	17 (3.1)
Household role		
Cooking	155 (28%)	155 (28%)
Babysitting	44 (8.0%)	44 (8%)
Fetching water	261 (47.0%)	260 (47%)
Others	97 (17%)	98 (18%)
Primary caregiver		
Father	237 (42.5%)	237 (42.5%)
Mother	225 (40.4%)	225 (40.4%)
Brother/sister	39 (7.0%)	39 (7.0%)
Uncle/aunt	56 (10.1%)	56 (10.1%)

### HIV testing and known status among adolescents at baseline and endline surveys

There was an increase in the proportion of adolescents who self-reported that they had ever tested for HIV (p<0.001) from 315 (56.7) at baseline to 557 (100.0) at endline, and those who knew their HIV status also increased from 316 (56.7) at baseline to 398 (71.5) at endline HIV testing and known status disclosure at baseline and endline (table 2).

**Table 2:** HIV testing and known status among adolescents at baseline and endline surveys (n=557)

Indicator	Baselinen (%)	Endlinen (%)	McNemar’sp value
Ever tested for HIV/AIDS			
Yes	315 (56.7)	557 (100.0)	
No	241 (43.4)	0 (0.0)	<0.001
HIV status			
Known	316 (56.7)	398 (71.5)	
Unknown	241 (43.3)	159 (28.6)	<0.001

### Bivariate associations between sociodemographic variables and HIV testing and status disclosure variables at baseline

In bivariate analyses, the participant’s employment status and reading proficiency were the only sociodemographic characteristics that were significantly associated with known HIV status. The odds of unknown HIV status were 58%, cOR 1.58 (95% CI 1.06, 2.35) higher among unemployed participants compared with the employed, and about 62% 0.62 (95% CI 0.39, 0.97) lower among participants who could read a whole sentence compared with those who could not read any sentence (table 3).

**Table 3:** Bivariate analysis of HIV status and demographic characteristics at baseline

Indicator	Known	Unknown	cOR (95% CI)
	n (%)	n (%)	
Sex			
Female	163 (51.6)	138 (57.3)	1.00
Male	153 (48.4)	103 (42.7)	0.79 (0.57, 1.11)
Education			
None	5 (1.6)	2 (0.8)	1.00
Primary	235 (74.4)	192 (79.7)	2.04 (0.39, 10.64)
Secondary	76 (24.0)	47 (19.5)	1.54 (0.29, 8.29)
Age category			
0–14 years	181 (57.3)	147 (61.0)	
15–17 years	135 (42.7)	94 (39.0)	0.86 (0.61, 1.21)
Employment status			
Employed	255 (80.7)	175 (72.6)	1.00
Unemployed	61 (19.3)	66 (27.4)	1.58 (1.06, 2.35)*
Reading proficiency			
Can’t read at all	47 (14.9)	52 (21.6)	
Reads part sentence	100 (31.7)	74 (30.7)	0.67 (0.41, 1.10)
Reads the whole sentence	169 (53.4)	115 (47.7)	0.62 (0.39, 0.97)
Health rating			
Poor/fair	126 (39.9)	108 (44.8)	1.00
Good	173 (54.8)	117 (48.6)	0.79 (0.56,1.12)
Excellent	17 (5.4)	16 (6.6)	1.10 (0.53, 2.28)

* significant at p<0.05.

### Multivariate analysis of sociodemographic predictors of unknown HIV status

The odds of unknown HIV status were 49% lower at the endline vs baseline (aOR 0.51; (95% CI: 0.92, 0.67). Male participants had significantly lower odds of unknown HIV status compared with females (aOR 0.77 (95% CI: 0.60, 0.99), while respondents who were caretaken by a non-parent had lower odds of unknown HIV status compared with those under their parents’ care (aOR 0.59; 95% CI: 0.41, 0.84). The age of the respondents was not associated with awareness of their HIV status (aOR 1.05, 95% CI: 0.77, 1.43) (table 4).

**Table 4:** Multivariate analysis of sociodemographic predictors of unknown HIV status

Indicator	aORs	P value	95% Cl
Year			
Baseline	1.00	1.00	1.00
Endline	0.51	<0.001	0.51 (0.92, 0.67)
Sex			
Female	1.00	1.00	1.00
Male	0.77	0.041	0.77 (0.60, 0.99)
Age category			
10–14 years	1.00	1.00	
15–17 years	1.05	0.751	1.05 (0.77, 1.43)
Reading proficiency			
Can’t read	1.00	1.00	1.00
Can partly read	0.74	0.105	0.74 (0.51, 1.06)
Able to read the whole sentence	0.73	0.13	0.73 (0.49, 1.09)
Employment status			
Employed	1.00	1.00	1.00
Unemployed	1.36	0.04	1.36 (1.01, 1.82)
Caretaker			
Parent	1.00	1.00	1.00
Non parent	0.59	0.003	0.59 (0.41, 0.84)

aORsadjusted ORs

## Discussion

We observed higher rates of HIV testing and status disclosure at the endline survey compared with the baseline, with almost all the adolescents reporting having ever tested for HIV and knowing their serostatus. A related study examining the legal rights of minority males to take up HIV testing found that despite the legal capacity to consent to HIV testing without their parents, few had ever tested for HIV and those who had not disclosed their HIV status to their parents were unlikely to get tested.^21^ In South Sudan, where adolescents under 14 are not legally empowered to consent to HIV testing without parental approval, low testing rates are prevalent. This further underscores the importance of raising awareness of the importance of HIV testing and status disclosure among adolescents. The influence of healthcare providers on the uptake of HIV testing services in clinical settings among adolescents has also been highlighted. A study in Nigeria revealed 23.7% lower HIV testing rates among adolescents.^22^ The higher testing rates of HIV testing and status disclosure observed in our study could be associated with the delivery of multifaceted factors, including increased awareness about HIV prevention, making them seek HIV testing. The negative association of the male gender to lower HIV testing and status disclosure is in line with the general country data where higher levels of stigma and limited social support services have been observed. A related study in Kampala, Uganda also revealed lower HIV testing among young transactional sex men.^23 24^ The lower disclosure could be associated with a high level of stigma, and lack of social support has also been identified as a barrier to status disclosure among adolescents in Eastern Africa.^25^ Equally, our approach to empowering caregivers and peer educators to encourage adolescents to seek HIV testing and know their HIV status could have contributed to the findings. Many studies conducted on adolescent self-disclosure of HIV testing and status revealed varied findings. A global systematic review of self-status disclosure showed an association between increased HIV testing and self-disclosure and higher awareness of HIV prevention among adolescents.^26^ While higher self-disclosure of HIV status was observed, it was found that stigma was the major barrier to self-disclosure among adolescents. In contrast with the high level of HIV testing and status disclosure found in our study, a study in Eswatini found a very low prevalence of HIV status disclosure among young adolescents, while older adolescents were more confident in disclosing their status.^27^ This difference could be attributed to the knowledge difference where adolescents in our study were more well-empowered with knowledge than those in Eswatini.

In Tanzania^28^ and Uganda,^29^ where the stigma associated with the disclosure of HIV status to family members was found to be high, the majority of the adolescents opted to self-disclose their status to family members as opposed to non-family members.^30^ Another study investigating the incentive to either disclose or withhold their HIV serostatus after testing among South African adolescents to their sexual partners revealed a 36% delayed disclosure for up to 1 year for fear of abandonment, discrimination, infidelity accusations and intimate partner violence.^31^ Unlike these studies, our findings showed high levels of HIV testing and disclosure among adolescents, possibly due to the disclosure counselling support provided to their caregivers, peers and healthcare providers. The risk of intimate partner violence as a result of HIV status disclosure has been raised as a concern in previous studies.^32^ In line with these findings, our study calls for greater engagement of caregivers, healthcare providers and adolescents themselves to be empowered to take up HIV testing and disclose their serostatus without fear of violence or discrimination.

## Conclusions

Male adolescents living under the care of either siblings or other relatives were less likely to get tested and know their HIV status. Our findings underscore the importance of targeting efforts towards these categories of adolescents in postconflict and resource-limited settings like South Sudan while at the same time focusing on peer-led HIV education interventions in conflict-affected settings.

## supplementary material

10.1136/bmjopen-2024-094008 online supplemental file 1

10.1136/bmjopen-2024-094008 online supplemental file 2

10.1136/bmjopen-2024-094008 online supplemental file 3

10.1136/bmjopen-2024-094008 online supplemental file 4

## Data Availability

Data are available in a public, open access repository. All data relevant to the study are included in the article or uploaded as supplementary information.
