# Supplementary material for: Predictors of HIV testing and status disclosure among young adolescents in postconflict settings: findings from a pre–post study design in Nimule per-urban town of South Sudan
Source: BMJ Open. 2025 Apr 2;15(4):e094008. doi: 10.1136/bmjopen-2024-094008 (PMC11966997; doi:10.1136/bmjopen-2024-094008)
Supplement: online supplemental file 3 [file bmjopen-15-4-s003.pdf]

## Appendix 3: Information and consent form

**Title of research**: Evaluating the link between Orphans and Vulnerable children programming and HIV prevention and Treatment Outcomes for adolescents impacted by HIV and AIDS in Nimule Town, South Sudan

**Names and affiliation of team members**: This study is led by Mr. Samuel Bojo as the principal investigator and in partnership with Agency for Research and Development Initiative (ARDI), a national organization in South Sudan, South Sudan Ministry of Health represented by Dr. Alexander Dimiti as the co-Investigator. Other members of the research team include Dr. Ambrose Agweyu and Prof. Gilbert Kokwaro as supervisors from Strathmore University Nairobo Kenya and Prof. Lucie Cluver Accelerate Hub led by University of Oxford and University of Cape Town as well as Ms. Jacqueline Poni from the UNDP Accelerator Lab in South Sudan

**Funding Source**: This study is mainly funded by the Accelerate Hub led by University of Oxford (R53899/ARDI), DAAD in-region PhD scholarship, University of Cape Town and UNDP South Sudan Accelerator Lab.

**Purpose(s) of research**: The purpose of this study is to collect your information regarding age, sex, education, marital status, employment, income, health status, knowledge of HIV prevention, violence, HIV risk intensions, access to HIV testing and treatment and use this information to evaluate the link between the program and HIV prevention and treatment outcomes for adolescents. This will help the program to understand how important this programme is and how it can be used to improve HIV prevention and treatment outcomes for adolescents in your community and beyond.

**Study procedures**: The study has recruited a total of 768 adolescents from 400 households for people living with HIV and AIDS. These households were identified from Nimule will be identified from Nimule Hospital ART clinic. These households will be followed 12 months during the study period. Adolescents and primary caregivers will receive targeted interventions including but not limited to quarterly cash transfer and financial literacy training, positive parenting training, HIV risk education, supportive home visits and referrals for HIV testing and treatment services in health facilities. Using a standardized questionnaire, information on age, sex, marital status, education, employment, income, access to HIV testing and treatment will be collected at baseline and end line. These data will be analyzed to evaluate the impact of the program on HIV prevention ( knowledge on HIV prevention, acquiring HIV testing and knowing own status, reduction in HIV risk behaviours) and treatment outcomes ( retention in treatment and viral load suppression) for adolescents before and after the study. You have been identified as important participant in this study if you so wish to be included. It is your right at any time during the study refuse to continue in the study. Your refusal will not affect you/your child (ren)'s participation or eligibility to receive services from the study.

**Expected duration of research and of participants' involvement**: This study will be conducted for a period of 18 months with 3 months of conducting base line survey and another 3 months of conducting end line survey. Participants are required to complete the study and provide information required during these surveys. In addition, participants will be assigned to intervention groups ( SIL|C groups, Positive parenting group and Health clubs). While in these groups, participants will complete one-week financial literacy training, receive quarterly cash transfer as business seed capital and complete 3- months of positive parenting. Your will receive HIV risk screening tools and those identified high risk will be supported and referred to acquire an HIV.STIs testing in the health facility. You will also be required to schedule and participate mandatory monthly home visit conducted by trained case care workers and this will not last for more than one hour in each such visits. To ensure that you have time for other things in your households, you will be involved in planning of these activities with the case care Workers.

**Risk**: Participation in this study doesn't not present significant risk of harm to you and your adolescents. However, they may be instances when a breach of confidentiality and your privacy may occur. This breach of confidentiality is likely to occur during collection of information on HIV testing, disclosure of HIV status (Objective 3) and viral load results (objective 4) which give the principal investigator and data collectors access to your sensitive information. In addition, asking sensitive questions such as ["have you ever had penetrative sex? "Has any member of your family died?] will increased the risk of psychosocial distress. In addition, recruiting PLHIV in the intervention groups may pose potential stigma and discrimination from community members which will in turn result to psychological trauma. To mitigate the occurrence of these risks and their potential harm, a component risk analysis has been developed. In addition, unique identify codes to identify you and your household will be assigned to all participants. All your personal data collected will be anonymized to concealed you identify. In addition, Research Assistants involved in the study will sign non-disclosure agreement.

To cope up with psychological distress experienced during the study period, the study will train Case Workers on psychosocial support counselling and respond to support any participant experiencing any psychological trauma during the study period. The study has also designed and incorporated playful sessions such as storytelling, singing, dancing, games and sports activities. During home visits and interview with children and adolescents, the case Workers with your consent will talk to your children in privacy. In addition to this, whenever, need be, CCWs will support and refer you and your children to a specialized counselor psychosocial counselor.

**Costs of participation in the study**: There will be no financial cost in participating in the study. The cost to you is mainly related to the time you will spend in completing the various interventions such as financial literacy training, completing the 14 modules of positive parenting classes, health education sessions and completed facility appointments

**Benefit:** By participating in the study, your household will eligible to benefit from the planned intervention of the study. These among others include quarterly cash transfer worth of $50 for 12 months conditioned on completing a one-week financial literacy and business plan, completing positive parenting training, receive health education sessions on HIV and AIDS and being supported to complete health facility referrals. By completing these trainings, you will increase your knowledge and skills on income generation, raise up your children in a non-violent way and access HIV services.

**Confidentiality:** necessary measures will be taken to protect confidentiality of the information collected from you and your family. These measure among other include: -

- The study has adopted a 9-digit alphanumeric Unique Identifier Coded (UICs) to de-identify adolescents and caregiver. During sharing of data via email, all names of study participants were removed and only UIC used.
- In addition, a non-disclosure agreement has been signed between research assistants and the study prior to engagement with study participants. This agreement reminds and emphasize all research assistants the importance of the principles of privacy and confidentiality as well as consequences of breach of this principle.
- During baseline and end line household survey, interviews with both caregivers and are held in privacy where adolescents interviewed in absence of the caregiver and vice versa
- Safe and secure storage of data: The study has put in place measure to restrict access to data collected from study participants. These among others include keeping all records in lockable shelves and access to record files was strictly under the M & E officer and Principal investigator. In addition, participant data entered in excel database was stored in M & E and principal investigator computers and password protected

**Voluntarism**: Participation in this study is entirely voluntary. You are free to decide if you want your child (ren) to be included or not. If you do agree to participate, you are free to change your mind at any time. If you do not want to participate in this study, there will not be any adverse consequences for you or your family.

**Consequences of participants' decision to withdraw from research:** Please note that some of the information that has been obtained about you/your child (ren) before the withdrawal may still be used in reports and publications if you do not specifically request that such be deleted. Overall, the researchers promise to make a good faith effort to comply with your wishes for privacy and confidentiality.

**What happens to research participants when the research is over**: Findings from this research will be shared with South Sudan MoH, University of Oxford, Strathmore University, donors and implementing partners. The researchers cannot guarantee that you will be informed of the results personally, however the final research report and summary brief of the work shall be made available both in hard and soft copies. If you have any questions about your participation in this research, you can contact the Principal Investigator through the contact details below:

Name: Samuel Bojo: Phone: +211 920700083; +211916726783, E-mail: bojolokien@gmail.com

**Adolescent Assent Documentation:** Here, the caregiver will consent on behalf of the adolescent by accepting the interviewer to administer interviews to all adolescents (10-17) in the household**.** If you are willing to participate in this research, please state this so that the interviewer can make note of this by appending that you have agreed to participate in the research

Do you assent to have your information included in the study?

Yes ☐ No ☐ If no, interviewer thank the adolescent. If Yes, Interviewer to complete the section below. I have fully explained this research to ______________________________________ and have given sufficient information, including about risks and benefits, to make an informed decision. I hereby affirm that ______________________________________ has assented and volunteered to be a participant in the research.

DATE: ______________________ SIGNATURE: ____________________NAME: ______________________________

PLEASE KEEP A COPY OF THE SIGNED INFORMED CONSENT.
