# Supplementary material for: Predictors of HIV testing and status disclosure among young adolescents in postconflict settings: findings from a pre–post study design in Nimule per-urban town of South Sudan
Source: BMJ Open. 2025 Apr 2;15(4):e094008. doi: 10.1136/bmjopen-2024-094008 (PMC11966997; doi:10.1136/bmjopen-2024-094008)
Supplement: Supplementary file 5 [file bmjopen-2024-094008.reviewer_comments.pdf]

PEER REVIEW HISTORY

BMJ Open publishes all reviews undertaken for accepted manuscripts. Reviewers are asked to complete a checklist review form and are provided with free text boxes to elaborate on their assessment. These free text comments are reproduced below.

ARTICLE DETAILS

Title (Provisional)

PREDICTORS OF HIV TESTING AND STATUS DISCLOSURE AMONG YOUNG ADOLESCENTS IN POST-CONFLICT SETTINGS: FINDINGS FROM A PRE-POST STUDY DESIGN IN NIMULE PER-URBAN TOWN OF SOUTH SUDAN

Authors

Bojo, Samuel; Kokwaro, Gilbert; Agweyu, Ambrose

VERSION 1 - REVIEW

**Reviewer**	**1**
**Name**	**Botelho, Eliã**
**Affiliation**	**Universidade Federal do Pará, Programa de Pós Graduação em Enfermagem**
**Date**	**24-Jan-2025**
**COI**	**None**

I really enjoyed reading your paper. I just have only few suggestions found bellow:

Abstract

Define the research question.

Introduction:

1) In line 46 define PEFPAR;

2) It would be great if the authors provide how many young people leave with HIV in Sudan;

3) The authors should clarify the research question;

Methods:

Baseline to end line. What do you mean?

Some suggestions to Settings: Is health education against HIV promoted in schools? Is there any policy specific for this? Are the HIV services (preventive, diagnosis and treatment) freely accessible? Are these services centralized to specialized centers? Cn these services be found in

public system? What about PrEP? Please, localize us in the HIV fight policies employed in your country;

Line 110 - IHV?

Variables should be better defined. The authors describe the outcomes and only it. Detail about the sociodemographic ones.

Results:

OK!

Discussion.

OK

Conclusion OK

**Reviewer**	**2**
**Name**	**de Walque, Damien**
**Affiliation**	**The World Bank, Development Research Group**
**Date**	**24-Jan-2025**
**COI**	**None**

While the topic and the setting of the study are interesting, I found that critical elements in the analysis were missing. Here are my comments and recommendations:

1) Introduction: Please state clearly the research question

2) Outcome: HIV status disclosure: Please clarify disclosure to whom? Anyone? Sexual partner? Parents?

3) While the authors declare to have a quasi-experimental study sign, this is, in my understanding, a pre-post comparison with no (non-randomized) control group allowing an evaluation of the counterfactual. I have little confidence in results from pre-post comparison as it does not allow accounting for other trends and events occurring between the baseline and the endline.

Other comments:

a) In my opinion, the section on data management is too long and could be reduced.

b) I noted a few mistakes in the abstract:

a. Background, 1st sentence, I suppose that “compressive” should be replaced by “comprehensive”.

b. The numbers in the results section of the abstract do not seem to add up. 557 is not 100% of 577 and 556 is not 99.8% of 557. Further inspection of the results section in the page, suggests that “577” in the results section of the abstract is incorrect and should be replaced by 557.

VERSION 1 - AUTHOR RESPONSE

Reviewer: 1
Dr. Eliã Botelho, Universidade Federal do Pará
Comments to the Author:
I really enjoyed reading your paper. I just have only few suggestions found bellow:

Abstract
Define the research question. For adolescents who participated in health education delivered by peer educators, do they report higher HIV testing rates and status disclosure after completing the intervention as compared to when they were at baseline? This has been included in the introduction section

Introduction:
1) In line 46 define PEFPAR; This had been defined
2) It would be great if the authors provide how many young people leave with HIV in Sudan; This has been added in the introduction section
3) The authors should clarify the research question; This has been clarified above.

Methods:
Baseline to end line. What do you mean? Here, we implied that two surveys were conducted between baseline and endline
Some suggestions to Settings: Is health education against HIV promoted in schools? Is there any policy specific for this? Are the HIV services (preventive, diagnosis and treatment) freely accessible? Are these services centralized to specialized centers? Cn these services be found in public system? What about PrEP? Please, localize us in the HIV fight policies employed in your country; South Sudan has adapted comprehensive sexuality education in schools and this has been widely accepted to enhance knowledge on HIV among school children. Due to high stigma, HIV services are not freely accessible, especially among young adolescents. Moreover, PrEP services have recently been introduced in key population programs and they are not widely accessible. This context has been added in the introduction section

Line 110 - IHV? This has been corrected to mean HIV
Variables should be better defined. The authors describe the outcomes and only it. Detail about the sociodemographic ones. The main socidemos (age, gender, education

and type of caregiver) have been included
Results:
OK!
Discussion
. OK
Conclusion OK

Reviewer: 2
Dr. Damien de Walque, The World Bank
Comments to the Author:
While the topic and the setting of the study are interesting, I found that critical elements in the analysis were missing. Here are my comments and recommendations:
1) Introduction: Please state clearly the research question. This has been defined
2) Outcome: HIV status disclosure: Please clarify disclosure to whom? Anyone? Sexual partner? Parents? This has been clarified to mean any trusted person (caregiver, sibling or peer)
3) While the authors declare to have a quasi-experimental study sign, this is, in my understanding, a pre-post comparison with no (non-randomized) control group allowing an evaluation of the counterfactual. We implied that this is a pre-post evaluation study design with a baseline acting as a control group for the endline survey participants.

I have little confidence in results from pre-post comparison as it does not allow accounting for other trends and events occurring between the baseline and the endline. In our binary logistic regression analysis, we used baseline and endline covariates; hence the rigour of our analysis and results
Other comments:
a) In my opinion, the section on data management is too long and could be reduced. We have reduced this section accordingly
b) I noted a few mistakes in the abstract:
a. Background, 1st sentence, I suppose that “compressive” should be replaced by “comprehensive”. This has been corrected
b. The numbers in the results section of the abstract do not seem to add up. 557 is not 100% of 577 and 556 is not 99.82% of 557. Our calculation shows that 556/557*100 = 99.82%. Further inspection of the results section in the page, suggests that “577” in the results section of the abstract is incorrect and should be replaced by 557. The 577 was a type with the 557 as the correct value. This has been revised.

Reviewer: 1
If you have selected ‘Yes’ above, please provide details of any competing interests.: not applicable
Reviewer: 2
If you have selected ‘Yes’ above, please provide details of any competing interests.: Not applicable
